# Insights into the Lignocellulose-Degrading Enzyme System Based on the Genome Sequence of *Flavodon* sp. x-10

**DOI:** 10.3390/ijms26030866

**Published:** 2025-01-21

**Authors:** Bao-Teng Wang, Shuang Hu, Dong Nyoung Oh, Chun-Zhi Jin, Long Jin, Jong Min Lee, Feng-Jie Jin

**Affiliations:** 1 College of Ecology and Environment, Nanjing Forestry University, 159 Longpan Road, Nanjing 210037, China; wbt@njfu.edu.cn (B.-T.W.); shuanghu@njfu.edu.cn (S.H.); isacckim@alumni.kaist.ac.kr (L.J.); 2 Department of Biotechnology, Pukyong National University, 45 Yongso-ro, Nam-gu, Busan 48513, Republic of Korea; ody3784@pukyong.ac.kr; 3 Cell Factory Research Centre, Korea Research Institute of Bioscience & Biotechnology (KRIBB), Daejeon 34141, Republic of Korea; chunsik@kribb.re.kr

**Keywords:** *Flavodon* sp., whole-genome sequencing, lignocellulose degradation, PCWDEs

## Abstract

The efficient hydrolysis of lignocellulosic biomass relies on the action of enzymes, which are crucial for the development of economically feasible cellulose bioconversion processes. However, low hydrolysis efficiency and the inhibition of cellulase production by carbon catabolite repression (CCR) have been significant obstacles in this process. The aim of this study was to identify the patterns of cellulose degradation and related genes through the genome analysis of a newly isolated lignocellulose-degrading fungus *Flavodon* sp. x-10. The whole-genome sequencing showed that the genome size of *Flavodon* sp. x-10 was 37.1 Mb, with a GC content of 49.48%. A total of 11,277 genes were predicted, with a total length of 18,218,150 bp and an average length of 1615 bp. Additionally, 157 tRNA genes responsible for transporting different amino acids were predicted, and the repeats and tandem repeats accounted for only 0.76% of the overall sequences. A total of 5039 genes were annotated in the Kyoto Encyclopedia of Genes and Genomes (KEGG) database, representing 44.68% of all genes, and 368 metabolic pathways were involved. Of the 595 genes annotated in the carbohydrate-active enzyme (CAZy) database, 183 are associated with plant cell wall-degrading enzymes (PCWDEs), surpassing those of *Aspergillus niger* (167), *Trichoderma reesei* (64), and *Neurospora crassa* (86). Compared to these three fungi, *Flavodon* sp. x-10 has a higher number of enzyme genes related to lignin degradation in its genome. Transporters were further identified by matching the whole-genome sequence to the Transporter Classification Database (TCDB), which includes 20 sugar transporters (STs) closely linked to sugar utilization. Through the comprehensive exploration of the whole-genome sequence, this study uncovered more vital lignocellulase genes and their degradation mechanisms, providing feasible strategies for improving the strains to reduce the cost of biofuel production.

## 1. Introduction

The volatility of oil prices affects the energy security and economic growth of countries as oil consumption continues to rise. As a viable alternative energy source, ethanol has garnered increasing attention [1]. The primary raw material for commercial ethanol production is grains, such as corn [2], in which the main starch components are converted into ethanol through fermentation [3,4]. However, with a growing global population and food shortages, the cost of producing ethanol needs to be reduced, for example, cheaper non-grain raw materials such as plant cell wall polysaccharides, mainly cellulose and hemicellulose, can be utilized for ethanol production. These plant cell wall polysaccharides are interconnected with each other and with lignin—an aromatic polymer—forming lignocellulose that provides structural stability and mechanical strength to plant cells. This also leads to the fact that these plant cell wall polysaccharides are difficult to degrade, thus requiring synergistic action from multiple enzymes [5].

Carbohydrates, as the most important and extensively distributed organic compounds in nature, serve as the cornerstone of material transformation and energy flow across the whole ecosystem [6]. Cellulose is the most widely distributed and most abundant polysaccharide in nature, accounting for more than 50% of the carbon content in the plant kingdom. In general wood, cellulose accounts for 40~50%, and 10~30% of hemicellulose and 20~30% of lignin. Cellulose is the main structural component of the plant cell wall and is often combined with hemicellulose, pectin, and lignin [7]. To date, studies have demonstrated that a variety of complex enzymes, mostly carbohydrate-active enzymes, are involved in the degradation of carbohydrates, with a wide variety of degradation enzymes targeting different types of carbohydrates. Carbohydrate-active enzymes (CAZys) are categorized into six groups based on their catalytic activity and structural properties: glycoside hydrolases (GHs), polysaccharide lyases (PLs), glycosyltransferases (GTs), carbohydrate esterases (CEs), carbohydrate-binding modules (CBMs), and enzymes with auxiliary activities (AAs). Within lignocellulose-degrading enzymes, cellulolytic enzymes are primarily classified into the families CBM1, AA9, AA3, and GH (5, 6, 7, 12, 45) [8,9,10,11,12], while lignin-degrading enzymes are mostly included in the AA (1–8) families [13,14,15,16,17,18,19,20]. Complex hemicellulases mainly comprise the families GH (10, 11, 26, 27, 31, 35, 36, 43, 53, 54, 93) and CE (1, 2, 3, 5, 12, 15, 16) [21,22,23,24,25,26,27], while pectolytic enzymes are more abundant in the families GH (28, 78, 95, 105), PL (1, 3, 10), and CE8 [28,29,30,31,32,33], which are collectively referred to as plant cell wall-degrading enzymes (PCWDEs). Some important industrial strains, such as *A. niger*, *T. reesei*, and *N. crassa*, have been utilized for cellulase production. According to the CAZy database [34], the numbers of PCWDE genes in *Aspergillus niger*, *Trichoderma reesei* [35], and *Neurospora crassa* [36] are 167, 64, and 86, respectively. *A. niger* is an excellent pectinase-producing strain [37] that can adapt to a variety of living environments, from plant surfaces to roots and seeds, due to its well-balanced gene content of diverse enzymes. Currently, the majority of lignin-degrading microorganisms are white rot fungi capable of secreting manganese peroxidase, lignin peroxidase, and laccase [38]. As a white rot fungus, *Flavodon flavus* is an excellent aromatic compound-degrading fungus that has been extensively used to treat dye waste [39].

*Flavodon* is a genus of *Basidiomycota* in the current taxonomy, mainly distributed in forests across Europe, Asia, and the Americas. This genus exhibits a high cellulase production capacity and plays a crucial role as one of the primary litter-degrading microorganisms in forest ecosystems. *Flavodon* spp. have been studied systematically since 1975, with an emphasis on the morphological observation and description of the genus using microscopes for straightforward taxonomic identification [40]. Microscopically, they are characterized by a hyphal system that is monomitic, rarely dimitic, and most hyphae are without clamp connections; cystidia are usually absent and spores thin-walled, smooth and hyaline. All species are saprobes, growing on decayed wood, and most of them are associated with white rot [41]. Until 1999, the description of *F. flavus* greatly promoted the study of lignin-degrading enzymes. This species may release a large number of quantities of extracellular lignin-modifying enzymes (LMEs) such as manganese-dependent peroxidase (MNP), lignin peroxidase (LIP), and laccase [42]. These enzymes hold immense potential for applications in various biotechnological fields, including the conversion of lignocellulosic biomass into feed, fuels, and chemicals; biobleaching of paper pulp; decolorization and detoxification of wastewater from sulfate bleach plants; as well as degradation of highly toxic environmental chemicals, such as dioxins, polychlorinated biphenyls (PCBs), various dye contaminants, and polyaromatic hydrocarbons [43,44]. Researchers have studied *F. flavus* and its metabolites using various molecular biology methodologies [45], with the most extensive research focusing on the use of wastewater treatment discharged from sugar refinery and paper mills. The species within this genus have been isolated from a variety of sources, including rotting coral seagrasses on the coast and dead trees in forests across a wide latitudinal range, from high-latitude cold coniferous forest to low-latitude tropical rainforests [46,47,48].

In addition to investigating waste pollutant remediation, this genus has also been discovered to have a strong litter-degrading capacity. Studies have shown that the *Flavodon* spp. have a symbiotic relationship with *Ambrosia beetles*, sparking interest in the further exploration of their symbiotic system. It has been found that each category of *A. beetles* has its own exclusive symbiotic fungi: *A. beetles* aids the fungus expand its spread range, while *Flavodon* sp. assists *A. beetles* in inhabiting decaying wood in the forest and avoiding competition from other insects [49].

Lignocellulosic biomass from agricultural crop residues, wood-abundant renewable resources that are becoming increasingly important as a future source of biofuels. Although the replacement of gasoline with cellulosic ethanol may substantially reduce greenhouse gases in the atmosphere and decrease global warming, the high cost of hydrolyzing biomass polysaccharides to fermentable sugars remains a major obstacle that must be overcome before cellulosic ethanol can be effectively commercialized. As the costs of cellulases and hemicellulases contribute substantially to the price of bioethanol, much cheaper sources of these enzymes are needed. Consequently, new studies aimed at understanding and improving cellulase efficiency and productivity are at the forefront of biomass research. To better understand this fungus and expand its extraordinary biotechnological potential has afforded impetus to the quest to sequence its genome. In this study, we isolated a new strain *Flavodon* sp. x-10 and conducted a whole-genome sequence analysis as well as a subsequent comparative genomic analysis, which will contribute to further investigations into the lignocellulose degradation mechanism of this fungus.

## 2. Results

### 2.1. Isolation, Screening, and Characterization of a High-Efficiency Cellulose-Degrading Fungus

**Figure 1:**
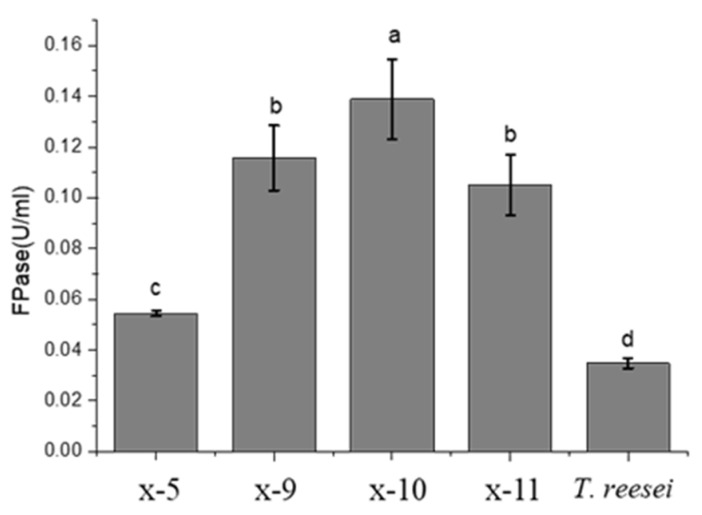
FPase activity of the isolates. After incubating in a CMC-Na liquid medium at 30 °C for 5 days, the FPase activity was determined of the isolates and reference strain *Trichoderma reesei*. Different lowercase letters represent significant (*p* < 0.05) differences between treatments (n = 3).

The samples collected from the litter of a poplar plantation were cultivated on a CMC-Na medium plate with serial dilution to isolate cellulose-degrading fungi. Following Congo red staining, strains with clear transparent zones around the colonies in Congo Red staining were chosen for further filter paper enzyme (FPase) activity assay (Figure 1). Subsequently, after incubation at 30 °C in a CMC-Na liquid medium for 5 days, the FPase activity of these isolates was determined and compared. Isolate x-10 exhibited the highest FPase activity (0.138 U/mL), which was significantly higher than those of other isolates and the reference strain *T. reesei* (0.0347 U/mL) (Figure 1). This finding was consistent with the result of Congo red staining on CMC-Na medium with the largest transparent circle produced by isolate ×10.

**Figure 2:**
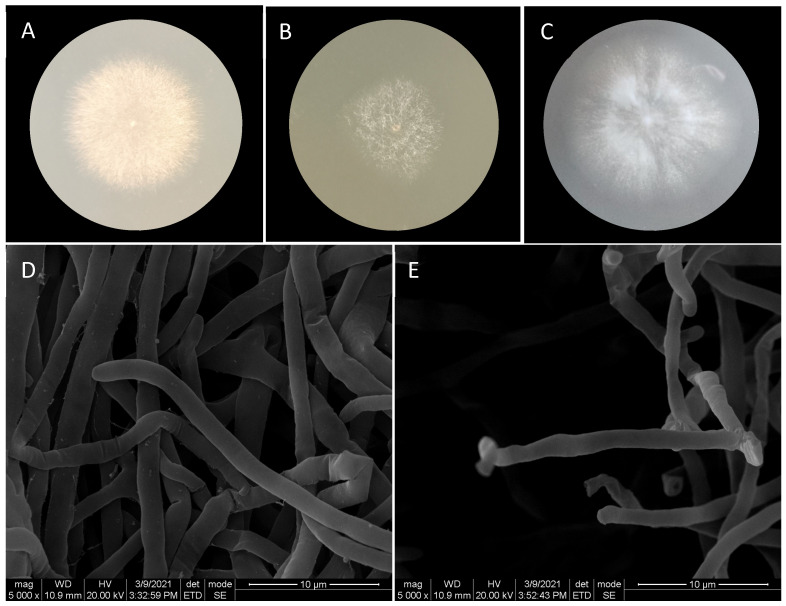
Colony morphology of *Flavodon* sp. x-10 on different media and the mycelium morphology under a scanning electron microscope (SEM). (**A**): Colony in the CYA medium cultured at 30 °C for 5 days, showing a large colony with sparse mycelia. (**B**): Colony in the PDA medium at 30 °C for 5 days; the colony was small. (**C**): Colony in the CMC-Na solid medium cultured at 30 °C for 5 days; mycelia were sparse. (**D**,**E**): The microscopic morphology of mycelia on the PDA medium cultured at 30 °C for 3 days ((**D**): SEM image magnified 5000 times; (**E**): SEM image magnified 5000 times).

**Figure 3:**
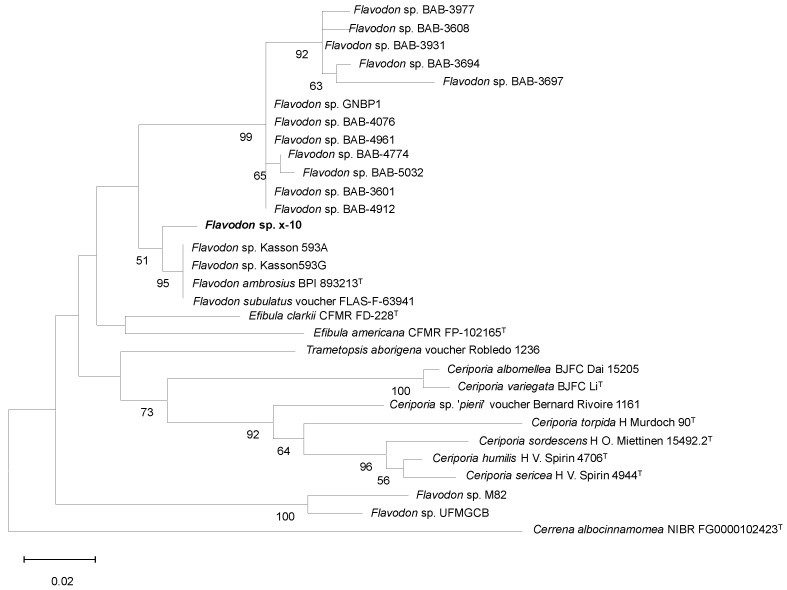
Phylogenetic tree of the genus *Flavodon* based on the ITS region. The phylogenetic relationship was inferred using the neighbor-joining method in MEGA 11. The percentage of replicate trees in which the associated taxa clustered together in the bootstrap test (1000 replicates) are shown under the branches. Evolutionary distances were computed using the Kimura 2-parameter method, and all positions containing gaps and missing data were eliminated. The T represents the type species.

The colony morphology of isolate x-10 on different media (PDA, CYA, and CMC-Na) exhibited consistent characteristics: transparent colony edges; white mycelium extending from the center to the edge; mycelium cells with a width of 1–2 μm, radially diffused hyphae without any presence of spores or other fruiting bodies (Figure 2). The phylogenetic analysis supported x-10 as a member of the genus *Flavodon* (Figure 3), and was named *Flavodon* sp. x-10. 

### 2.2. Basic Genome Information for Flavodon sp. x-10

According to the results of whole-genome sequencing, *Flavodon* sp. x-10 had a total of 7,998,389,159 raw bases, with 516,732 raw reads. The assembled genome was approximately 37.10 Mb in size, with a GC content of 49.48% and 46 contigs (Table 1). The completeness of the genome assembly of *Flavodon* sp. x-10 was assessed based on the BUSCO analysis. The results show that a total of 290 fragments were assembled, of which 277 were complete fragments, with 274 single copies and 3 multiple copies, indicating that genome assembly captured 95.51% of the complete BUSCO. The results suggest that the genome assembly has great integrity and reliability for further data analysis.

The gene prediction identified a total of 11,277 putative genes with an average length of 1615 bp each, totaling 18,218,150 bp and accounting for 49.11% of the whole-genome length, and the GC content in the gene region was 52.28%. The basic genome information for strain *Flavodon* sp. x-10 is shown in Table 1. The distribution analysis revealed that most of the genes were less than 3000 bp in length, while only about 18% of them were longer than 3000 bp (Figure S1).

### 2.3. Statistics of the Non-Coding RNA Genes

Differences in tRNA composition reflect the diversity in amino acid transport across organisms. In this study, a total of 157 tRNA genes were identified, with 153 responsible for transferring specific amino acids as indicated in Table 2. tRNA genes responsible for glycine and serine are the most abundant, while the number of tRNA genes responsible for cysteine are the least common, with the majority ranging between 4 and 10. Disparities in tRNA gene abundance for specific amino acids indicate a bias in the amino acid composition of the proteins for this strain. The results suggest that the requirements of alanine, arginine, glutamic acid, glycine, leucine, serine, and valine in the strain *Flavodon* sp. x-10 are relatively high, whereas aspartate, cysteine, and tryptophan are less required. In addition, there is little difference in the number of four distinct rRNA genes (5S, 12 genes; 5.8S, 11 genes; 18S, 11 genes; and 28S, 12 genes).

### 2.4. Repeat Sequence Analysis

In the genome of *Flavodon* sp. x-10, the repeat sequences consist of interspersed repeats and tandem repeats, which account for just 0.76% of the overall sequence, and the proportion was relatively low (Table 3). Among the repeats implicated in fungal spontaneous mutations, long interspersed nuclear elements (LINEs) had the largest number (60), followed by short interspersed nuclear elements (SINEs) (7) and long terminal repeat (LTR) elements (6). This suggests that spontaneous mutations of *Flavodon* sp. x-10 are primarily driven by LINEs. The low proportion of the repeats implies that *Flavodon* sp. x-10 has a relatively weak capacity for spontaneous mutation. These repetitive sequences carry a large amount of genetic information and are integral to gene regulatory network. They play an indispensable role in gene expression and transcription regulation, influencing the evolution and inheritance of and variation in life.

### 2.5. Functional Annotation

The functional gene annotation and classification of the whole genome of *Flavodon* sp. x-10 were conducted using the COG (Cluster of Orthologous Groups), GO (Gene Ontology), and KEGG databases, resulting in successful annotations of 2080, 10,429, and 5039 unigenes, respectively.

**Figure 4:**
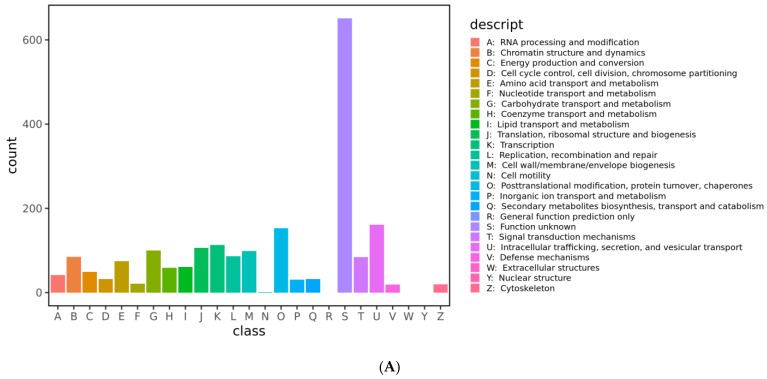

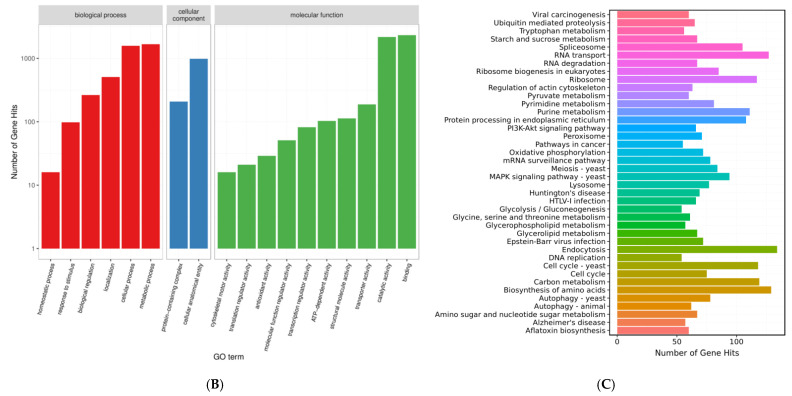
Functional annotations. (**A**) COG annotation and classification. (**B**) Annotation and classification of GO terms. (**C**) KEGG pathway functional annotation.

Based on COG analysis for gene annotation and functional classification, it was found that the 2080 unigenes closely matching the statistics in this database could be assigned to 23 categories (Figure 4A). Among them, carbohydrate transport and metabolism (G), translation, ribosomal structure and biogenesis (J), transcription (K), post-translational modification, protein turnover, chaperones (O), intracellular trafficking, secretion, and vesicular transport (U) were discovered to be the most abundant, accounting for 4.80%, 5.09%, 5.43%, 7.35%, and 7.74% of the total, respectively.

In three databases, the most genes in the *Flavodon* sp. x-10 genome were successfully annotated into the GO database and classified into three major functional groups: biological process, cellular component, and molecular function. Of the three, molecular function contained the highest number of functional categories and gene count (5072), followed by biological process (Gene No: 4136) and cellular component (Gene No: 1192) (Figure 4B). In terms of molecular function, the ATP binding (GO: 0005524; 702 genes), DNA binding (GO: 0003677; 218 genes), metal ion binding (GO: 0046872; 114 genes), zinc ion binding (GO: 0008270; 59 genes), and hydrolase activity (GO: 0016787; 130 genes) were the most abundant subcategories, with the first four belonging to the binding pathway. In addition, oxidation reduction process (GO: 0055114; 749 genes) and transcriptional regulation (GO: 0006355; 104 genes) were the most enriched subcategories in the biological process, while integral component of membrane (GO: 0016021; 427 genes) and cytoplasm (GO: 0005737; 54 genes) pathways accounted for a significant proportion of the cellular component.

Of the 11,277 predicted genes, 5039 were annotated in the KEGG database, representing 44.68% of all genes. A total of 368 metabolic pathways are implicated, and the distribution of genes involved in major metabolic pathways is illustrated in Figure 4C. These findings indicate that *Flavodon* sp. x-10 has the potential to participate in the metabolic processes associated with most carbon sources in its natural habitat, which aligns with the results of its carbon source utilization experiment (Table S1). Among them, genes involved in the endocytosis (map04144; 134 genes), biosynthesis of amino acids (map01230; 129 genes), carbon metabolism (map01200; 119 genes), yeast cell cycle (map04111; 118 genes), ribosome (map03010; 117 genes), purine metabolism (map00230; 111 genes), and protein processing in the endoplasmic reticulum (map00270; 108 genes) were the most abundant.

### 2.6. Major Lignocellulosic Degradation Processes

**Figure 5:**
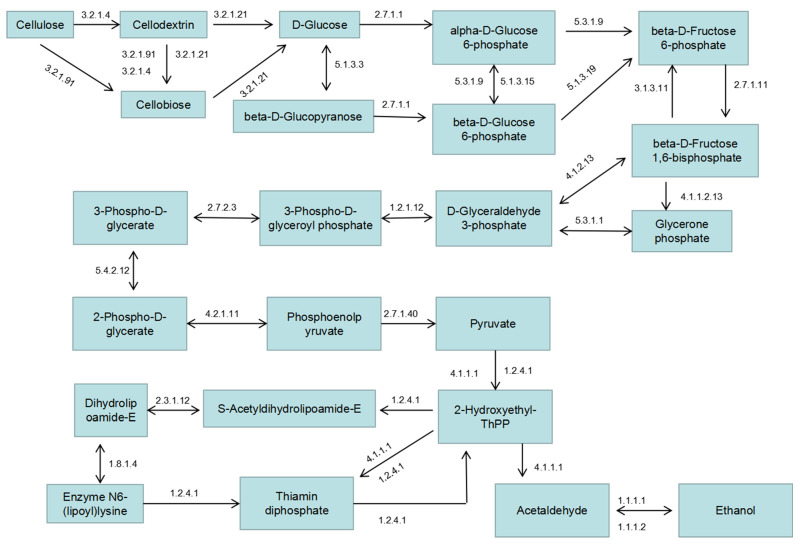
Cellulose-to-ethanol metabolic process based on the genomic analysis of isolate *Flavodon* sp. x-10. The reaction substrates or products are shown in the box. The arrows denote the direction in which the enzyme reaction proceeds. The numbers above the arrow represent the EC numbers of the enzymes; for example, 3.2.1.4 stands for EC 3.2.1.4.

Since *Flavodon* sp. x-10 has a high cellulose degradation capacity, we mainly focused on its carbon metabolic pathways related to lignocellulosic degradation, including the degradation of aromatic compounds, glycolysis/gluconeogenesis, and starch and sucrose metabolism (Table S2). In the metabolic profiles, the largest number of genes (50) were involved in the degradation of aromatic compounds across all metabolic pathways. These genes are involved in a total of 13 different metabolic processes and contain multiple oxidase and peroxidase genes that contribute to the degradation of lignin and provide various fatty acids, polysaccharides, and aldehydes for other metabolic pathways. The structure of lignocellulose is complex, which poses a major challenge to its utilization due to its difficult degradation. *Flavodon* sp. x-10 was isolated from litter and dead logs, and its genome was also found to contain a variety of genes related to the degradation of aromatic compounds. Another critical metabolic pathway annotated by KEGG is glycolysis and synthesis, which serves as the central metabolic pathway in a living system and is associated with some essential metabolic pathways in organisms, such as starch and sucrose metabolism, pentose phosphate pathway, citrate cycle, propanoate metabolism, and carbon fixation in photosynthetic organisms. In this process, various external polysaccharides are degraded into glucose, which is the substrate for the synthesis of other substances necessary for the life activities of the organism. The metabolic pathways from cellulose to ethanol are complete, including the entire degradation of cellulose to glucose within the starch and sucrose metabolism pathway, involving 22 enzymes (80 genes) (Table S3). By integrating various metabolic pathways associated with forest litter degradation, we can infer the decomposition process of forest litter by *Flavodon* sp. x-10. The metabolic pathway diagram from cellulose to ethanol in *Flavodon* sp. x-10 is depicted in Figure 5, while the enzymes involved in this metabolic pathway are listed in Table S3.

### 2.7. CAZy

**Figure 6:**
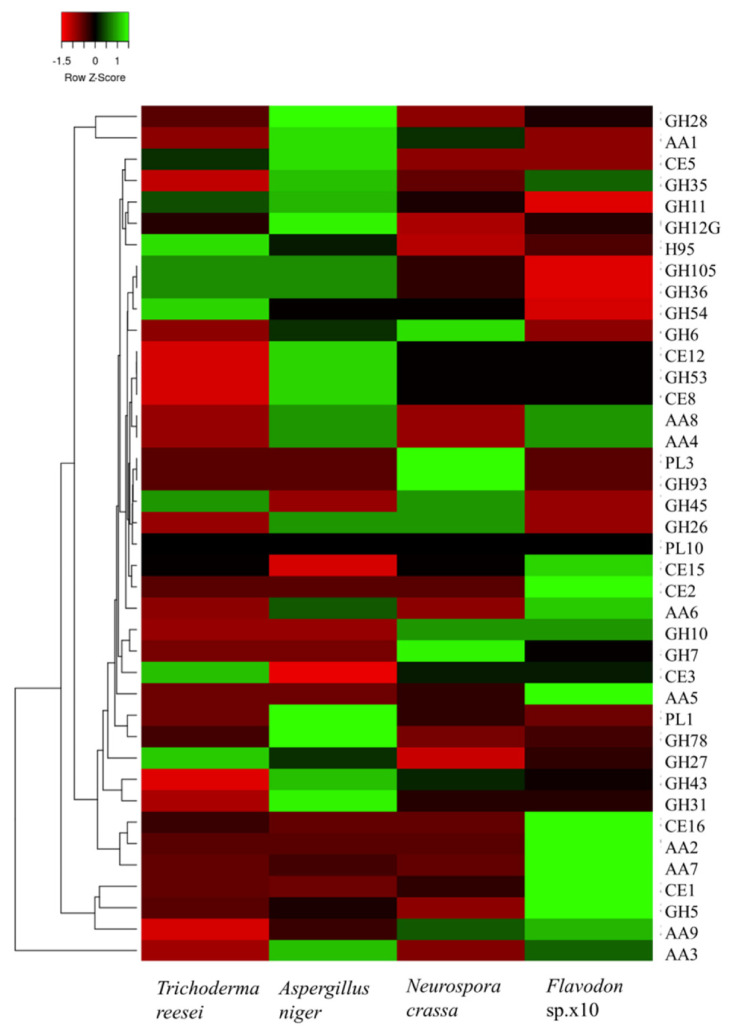
Heatmap comparison of PCWDE genes between *Flavodon* sp. x-10 and three other species.

The respective enzyme families of carbohydrate-active enzyme genes annotated to the *Flavodon* sp. x-10 genome were further analyzed. The heatmap analysis showed that, compared to the other three cellulose-degrading fungi, *Flavodon* sp. x-10 has a larger number of enzyme genes involved in lignin degradation (AA2, AA4, AA5, AA6, AA7, AA8, and CE16) and fewer pectin-degrading enzyme genes (GH28, GH78, GH95, GH105, PL1, PL3, PL10, and CE8) (Figure 6).

**Figure 7:**
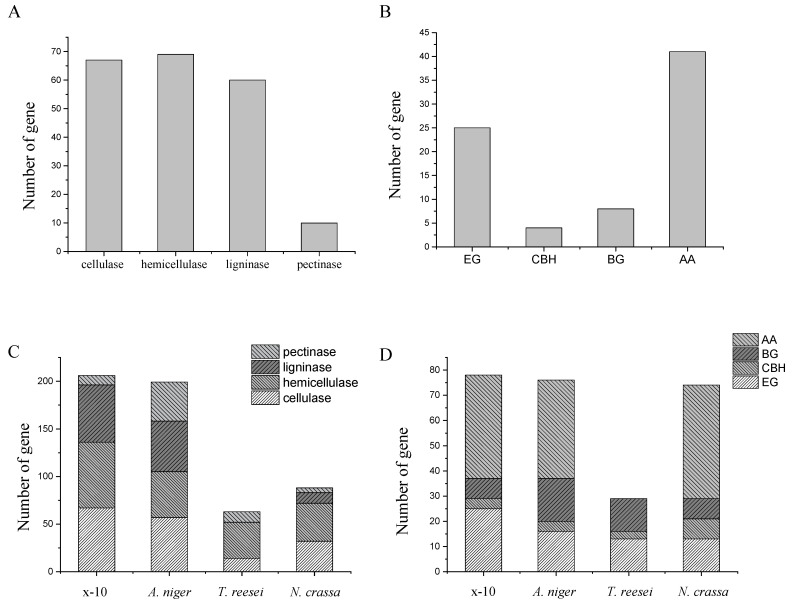
Comparison of the number of PCWDE and various cellulolytic enzyme genes. The number of PCWDE genes (**A**) and various cellulase genes (**B**) in the *Flavodon* sp. x-10 genome. Comparison of PCWDE (**C**) and cellulolytic enzyme (**D**) genes between *Flavodon* sp. x-10 and three other cellulase-producing fungi. EG, endoglucanase; CBH, cellobiohydrolase; BG, *β*-glucosidase; AA, auxiliary activity.

Compared to the other three cellulose-degrading fungi [50,51], *Flavodon* sp. x-10 had the most abundant enzyme genes related to lignin degradation, accounting for 29.13% of the whole lignocellulose-degrading enzyme genes, while the number of pectinase genes related to pectin degradation was relatively small, accounting for only 4.85% of PCWDE genes (Figure S2). The quantitative composition of PCWDE genes in the four species is shown in Figure 7.

**Figure 8:**
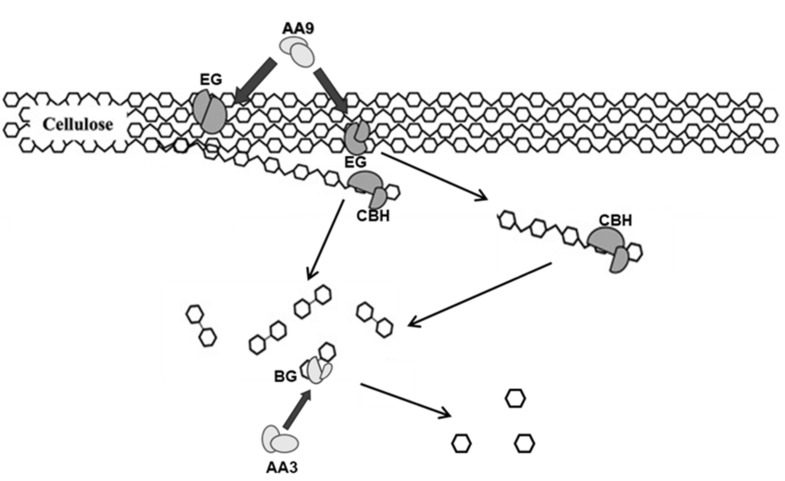
Action modes of various enzymes during cellulose degradation. In the process of cellulose degradation, AA9 assists EG to degrade cellulose into small fragments from the inside; CBH cuts small molecules, such as cellobiose, at both ends of cellulose; AA3 subfamily assists BG in hydrolyzing small molecular fragments, such as cellobiose, into glucose.

In the *Flavodon* sp. x-10 genome, the number of PCWDE genes was the most abundant (183), followed by *A. niger* (167). However, *T. reesei* [35] and *N. crassa* [36] had relatively fewer PCWDE genes, with 64 and 86 genes, respectively. The proportion of pectinase in *N. crassa* was 5.82%, similar to that in *Flavodon* sp. x-10. In addition, the proportion of lignin-degrading enzyme genes in *N. crassa* and *T. reesei* was much lower than that of *Flavodon* sp. x-10, suggesting that *Flavodon* sp. x-10 could potentially degrade lignocellulose better than the first two species. The number of PCWDE genes in *Flavodon* sp. x-10 was nearly 3 times that of *T. reesei*, and the number of genes associated with cellulose degradation was about 2.5 times that in *T. reesei* (Tables S4 and S5). Compared to *T. reesei*, *Flavodon* sp. x-10 had little difference in the number of endoglucanase (EG) and β-glucosidase (BG) genes, but it contained more auxiliary activity (AA) genes. The action modes of various enzymes during cellulose degradation are shown in Figure 8. This explains why the filter paper enzyme activity of *Flavodon* sp. x-10 is four times higher than that of *T. reesei* (Figure 1).

### 2.8. TCDB

In addition to the above, a total of 568 transporter genes, involving 105 transport systems, were annotated in TCDB. These encompass the largest number of transporter systems, such as the Major Facilitator Superfamily (MFS) (Table 4). There are 103 genes annotated in the MFS, which is the most annotated transport system in TCDB. As a saprophytic fungus, *Flavodon* sp. x-10 requires MFS for substance transport and extracellular enzyme secretion. Among these transporters, the STs play a key role in sugar utilization. The *Flavodon* sp. x-10 genome contains 20 ST genes (Table S6), fewer than that in *T. reesei* [52].

Other transporters are also mainly involved in the synthesis, transport, and catabolism of secondary metabolites, intracellular material transfer, secretion and membrane vesicle transport, and material transport of energy metabolism. These transport systems are closely related to the degradation of lignocellulose by *Flavodon* sp. x-10.

### 2.9. Different Cellulase and Laccase Activity

**Figure 9:**
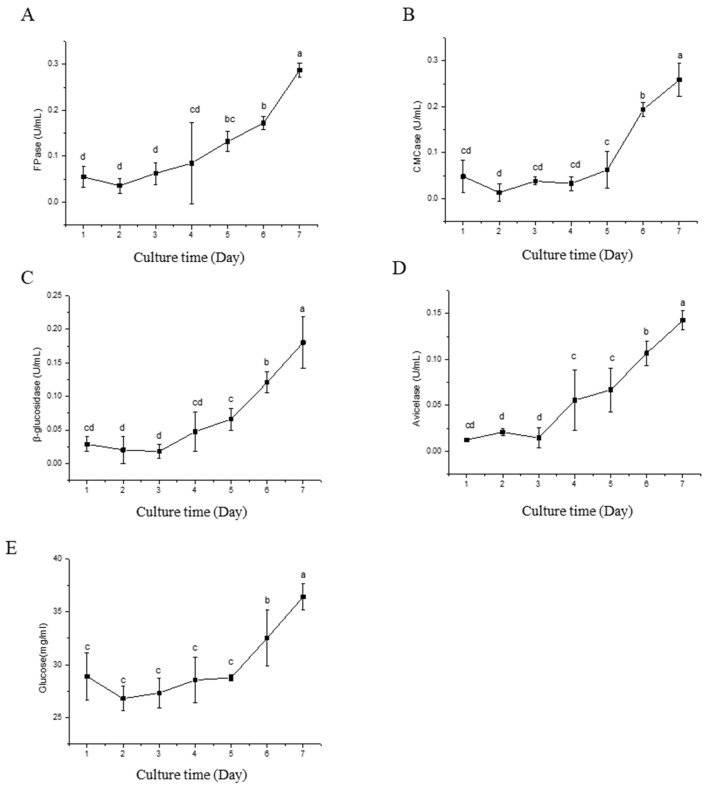
Changes of cellulase activity (**A**–**D**) and glucose content (**E**) in the CMC-Na liquid medium with different culture times. Different lowercase letters represent significant (*p* < 0.05) differences between treatments (n = 3).

The genomic analysis revealed that the *Flavodon* sp. x-10 genome harbors a higher number of cellulase and ligninase genes. To further explore the enzymatic properties of *Flavodon* sp. x-10, more detailed assessments of cellulase and laccase activities were conducted. Isolate x-10 was cultured in a CMC-Na liquid medium for 7 days, and the activities of various cellulose degradation-related enzymes and glucose content in the culture supernatant were measured with the culture time. The results show that the glucose content of the liquid medium remained relatively low during the initial 5 days, but exhibited a significant increase on the 6th day (Figure 9E). Correspondingly, these enzyme activities were relatively low in the early stage of culture, but showed substantial increases in the middle and late stages of culture (Figure 9).

**Figure 10:**
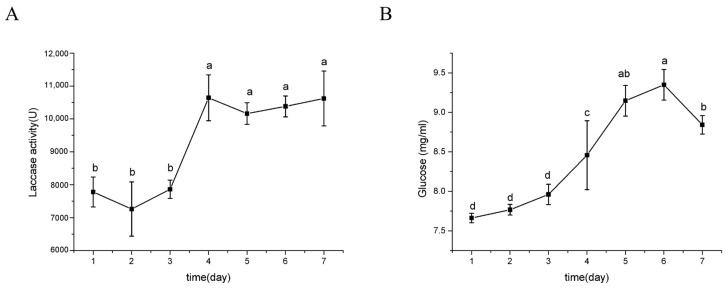
Changes in laccase activity (**A**) and glucose content (**B**) with culture time. Different lowercase letters represent significant (*p* < 0.05) differences between treatments (n = 3).

A lignin-containing liquid medium was utilized to induce and measure laccase activity (Figure 10). The glucose content in the medium remained low for the first 3 days, increased significantly from day 4 onwards, peaked on day 6, and then a decline on day 7. The laccase activity remained at a low level during the initial three days before experiencing a significant increase to reach its peak on the 4th day, subsequently maintaining high levels until day 7.

## 3. Discussion

In this study, the whole genome of a high-efficiency cellulose-degrading isolate *Flavodon* sp. x-10 was sequenced, assembled, and annotated using PacBio third-generation sequencing technology. Through comparative genomic analysis, the lignocellulosic degradation-related genes of *Flavodon* sp. x-10 were compared to those of three other cellulose-degrading fungi, *T. reesei* [53], *A. niger* [54], and *N. crassa* [20], to explore their differences in degradability and features. The sequencing analysis showed that the whole-genome size of *Flavodon* sp. x-10 was 37.10 Mb, located in the middle of *T. reesei* (33.40 Mb), *A. niger* (33.98 Mb), *N. crassa* (41.10 Mb).

According to the KEGG metabolic pathway, *Flavodon* sp. x-10 is apparently involved in a wide range of metabolic processes (Figure 4C) and demonstrates the ability to utilize a diverse range of carbon sources in its natural habitat, indicating its potential widespread distribution. However, despite its versatile metabolism, *Flavodon* sp. primarily survives in forest litter and does not constitute a dominant species [55], which reflects the niche selection of strains in this genus. Despite its capacity to use a variety of carbon sources (Table S1), the isolate x-10 is unable to outcompete others for certain carbon sources, as observed by comparing its growth with glucose versus cellulose as external carbon sources.

The CAZy annotation results show that, although the *Flavodon* sp. x-10 genome is not the largest, it has a much higher number of CAZy genes associated with lignocellulosic degradation than the other three cellulosic-degrading fungi (Figure 7) [35,36,37]. In contrast to the other three fungi, *Flavodon* sp. x-10 exhibits a greater abundance of genes in the AA2, AA3, AA4, AA5, AA7, AA8, and CE1 subfamilies, which are known to be primarily involved in lignin degradation (Figure 7C). Notably, the gene in the AA5 subfamily [56], a key enzyme for lignin degradation, exists in multiple copies within the *Flavodon* sp. x-10 genome, thereby contributing to its effective lignin degradation. Lignin provides support for the mechanical strength of plants, especially woody plants [57], and as one of the main components of lignocellulose, it is known as a complex organic phenol polymer that is challenging to degrade. Insect herbivores typically consume young branches and leaves with a low lignin content rather than directly feeding on xylem with a high lignin content [58]. However, they utilize another approach, for example, symbiotic interaction with *Flavodon* sp., a fungus capable of breaking down and utilizing lignocellulose, while insects feed on its mycelium [59]. The most extensively studied species within the genus *Flavodon* is *Flavodon flavus*, which naturally inhabits decaying seagrass in coastal mangroves [60] and felled, decayed bamboo [61]. *Flavodon flavus* is mostly employed to remove lignin in various industrial wastewater [45] and also to remove lignin during the extraction of cellulose in plants [42]. And despite having its entire genome sequenced, no related studies have been published. *Flavodon* sp. x-10 in this study was isolated from litters of poplar plantations near the coastline, and the living environment was not much different from that of *F. flavus*, with forest litter serving as the base for survival. Based on these findings, the study of the corresponding functional genes in the *Flavodon* sp. x-10 genome, especially its abundant genes related to lignin degradation, can be exploited to reduce the lignin pollution produced by paper mills.

A variety of transporters annotated in the TCDB database facilitate the transportation of diverse reaction substrates to support the complex metabolic activities of organisms (Table 4). Simultaneously, the diversity of carbohydrate activity enzymes enhances *Flavodon* sp. x-10’s adaptability to its environment. The *Flavodon* sp. x-10 genome contains relatively few ST genes (Table S6), with hexose transporter genes being the most numerous (16), followed by D-glucose (10), D-xylose (6), and cellobiose (5). These results demonstrate significant functional redundancy and complexity in sugar transport within strain *Flavodon* sp. x-10. The genome of strain x-10 typically harbors multiple STs with similar substrate specificity, as well as individual transporters capable of transporting different sugars [62]. For instance, within the *Flavodon* sp. x-10 genome, there are 5 ST genes (2018_t, 2019_t, 10393_t, 2165_t, and 6607_t) responsible for cellobiose transportation, among which the gene 1736_t encoding transporter can simultaneously transport D-glucose, hexose, and D-xylose.

The results of enzyme activity measurement indicate that the increase in glucose content resulting from cellulose degradation in the medium did not inhibit cellulase production (Figure 9), leading to a continuous rise in various cellulolytic enzymes during the later stages of culture. However, according to the carbon catabolite repression (CCR) mechanism, when there is a certain concentration of glucose in the external environment, the secretion of cellulase should be suppressed [63]. Interestingly, no homologous sequence of the key CCR transcription factor CreA/Cre1 was found in the genome of *Flavodon* sp. x-10. Similarly, the laccase activity results show that the glucose contained in the medium promoted the rapid growth of the strain during the early stage of culture, and subsequently, significant laccase secretion was induced by alkaline lignin in the middle and late stages of culture. The sustained increase in glucose concentration within the culture environment did not compromise the high laccase activity secreted by isolate x-10 (Figure 10). Although strain x-10 possesses a significantly lower quantity of ST genes (Table S6) compared to *T. reesei*, its cellulase activity is conspicuously higher than that of *T. reesei* (Figure 1). This is intimately associated with another function of STs: they play a role in sensing the ambient sugar levels and triggering corresponding intracellular signaling [64]. *Flavodon* sp. x-10 has a small number of ST genes; consequently, it is less susceptible to variations in the sugar concentration of the external environment than other species. This, combined with the absence of homologues of CreA—a key transcription factor for CCR—within the *Flavodon* sp. x-10 genome, leads to its capacity for efficient cellulase production even under environments with high glucose concentrations. The isolation environment for strain x-10 resembles that of other *Flavodon* sp. [43,44,45], all of which were isolated from lignocellulose-rich environments [46,47,48]. Among these, *F. flavus* is commonly utilized in paper mills and sewage treatment plants for the degradation of cyclic aromatic hydrocarbons. In conclusion, whole-genome analysis and enzyme activity determination revealed the strong lignocellulose degradation capacity of *Flavodon* sp. x-10, suggesting that the isolate holds significant application value and industrial enzyme production potential in the future.

## 4. Materials and Methods

### 4.1. Sampling Site and Culture Media

The sampling site was located in the poplar plantations (120°49′ E, 32°52′ N) of Huanghai Forest Park, eastern China, close to the Yellow Sea. The soil is sandy, desalted, and meadowy, with a slightly alkaline pH.

A carboxymethyl cellulose sodium (CMC-Na) agar medium (10.0 g/L sodium carboxymethyl cellulose, 3.0 g/L peptone, 0.2 g/L yeast extract, 2.0 g/L (NH_4_)_2_SO_4_, 4.0 g/L KH_2_PO_4_, 0.3 g/L MgSO_4_·7H_2_O, and 20.0 g/L agar) was used to screen cellulose-degrading fungi. The CMC-Na liquid medium (10.0 g/L CMC-Na, 8.0 g/L peptone, 0.2 g/L yeast paste, 2.0 g/L (NH_4_)_2_SO_4_, 4.0 g/L KH_2_PO_4_, 0.8 g/L MgSO_4_·7H_2_O, 20.0 g/L glucose, 0.01 g/L vitamin B1, 0.2 g/L CaCO_3_, and 0.3 g/L ZnSO₄·7H_2_O) and laccase-induced liquid culture medium (10.0 g/L glucose, 3 g/L lignin, 3.0 g/L KH_2_PO_4_, and 0.03 g/L vitamin B1) were used to determine cellulase and laccase activities, respectively. The potato dextrose agar medium (PDA; BD Difco, Sparks, MD, USA) and Czapek yeast autolysate agar medium (CYA; Yi Fei Xue Biotechnology, Nanjing, China) were used for phenotypic observation.

### 4.2. Isolation and Preliminary Screening of Cellulose-Degrading Fungi

The samples collected from the litter of a poplar plantation (120°49′ E, 32°52′ N) of Huanghai Forest Park, eastern China, close to the Yellow Sea, were cultivated on a CMC-Na plate with serial dilution to isolate cellulose-degrading fungi. Following Congo Red staining, strains with clear transparent zones around the colonies were chosen for further filter FPase activity assay (Figure 1). Subsequently, after incubation at 30 °C in a CMC-Na liquid medium for 5 days, the FPase activity of these isolates was determined and compared. The specific experimental methods are described as follows: The collected humus soil (1.0 g) was thoroughly mixed in a centrifuge tube with sterile water, and then diluted in a gradient series ranging from 10^−2^ to 10^−9^. A 300 μL suspension was spread onto CMC-Na agar medium and cultured in an incubator at 30 °C for 2–3 days, followed by staining with Congo Red for screening. The staining process is described as follows: The 0.1% Congo Red solution was poured onto the plate, covering the whole plate over all the colonies for 20 min. The Congo Red solution was removed from the plate and poured in a 1 mol/L NaCl solution to wash away any remaining Congo Red. Colonies with relatively large transparent circles were selected for further purification, culture, observation (environmental scanning electron microscopy (FEI Quanta 200; FEI Company, Hillsboro, OR, USA), and enzyme assay.

### 4.3. Determination of Enzyme Activities

#### 4.3.1. Cellulase Activity Assay

The selected fungi were grown in the CMC-Na liquid medium at 30 °C and 150 rpm for 7 d, with three replicates for each strain. The filtered supernatant was used to determine the enzyme activity.

A glucose standard curve was constructed by preparing a series of 1 mg/mL glucose standard solutions ranging from 0 to 1 mL, adding distilled water to reach a total volume of 1 mL in each calibration tube, followed by the addition of 2 mL boric acid buffer (pH 10) and 2 mL DNS reagent. After incubation in a boiling water bath for 30 min and cooling to room temperature, the OD value at 540 nm was measured using a Nanodrop 2000C spectrophotometer (Thermo Fisher Scientific, Waltham, MA, USA).

FPase: A substrate solution containing filter paper in citrate buffer (pH 4.5) was prepared at a concentration of 1%. A total of 1 mL supernatant enzyme solution was mixed into 2 mL substrate solution and incubated in a water bath at 50 °C for 30 min. Following the termination of the reaction, 2 mL DNS reagent and 2 mL boric acid buffer (pH 10) were added to 1 mL reaction solution successively, then subjected to boiling water bath treatment for another 30 min. The absorbance was measured at a wavelength of 540 nm. One unit of enzyme activity is defined as 1 μmol of glucose produced per minute.

EG: A substrate solution containing sodium carboxymethyl cellulose in citrate buffer (pH 4.5) was prepared at a concentration of 1%. A total of 1 mL of supernatant enzyme solution was mixed into 2 mL substrate solution and incubated in a water bath at 50 °C for 30 min. Following the termination of the reaction, 2 mL DNS reagent and 2 mL boric acid buffer (pH 10) were added to 1 mL reaction solution successively, then subjected to boiling water bath treatment for another 30 min. The absorbance was measured at a wavelength of 540 nm.

CBH and BG: Enzyme activities were determined using microcrystalline cellulose and salicylate (in citrate buffer, pH 4.5) as substrate solutions, respectively, under the conditions described above.

#### 4.3.2. Laccase Activity Assay

The fungus selected was cultivated in a laccase-induced liquid culture media at 30 °C and 150 rpm for 7 days, with three replicates for each strain. The filtered supernatant was utilized to determine the laccase activity.

2-20-azinobis-(3-ethylbenzothiazoline-6-sulfonic acid) (ABTS) [65] was employed to assess the activity of laccases secreted by isolate x-10 during lignin degradation. Subsequently, a mixture of 1.0 mL ABTS solution (0.5 mM) and 1 mL sodium acetate buffer (10 mM; pH 4.6) was incubated at 37 °C. Following this, the reaction was initiated by adding in 1 mL mixed culture supernatant. After a duration of 10 min, absorbance at a wavelength of 420 nm was directly measured using a Nanodrop 2000C spectrophotometer (Thermo Fisher Scientific, Waltham, MA, USA). The micromoles of oxidized ABTS were calculated using an Σ420 value of 36,000 (mol oxidized ABTS)^−1^cm^−1^. The unit (U) of enzyme activity is defined as the amount of enzyme required to change the absorbance at 420 nm by 0.001 per minute at 37 °C. The formula is as follows:

$$U(U/L)=(∆OD420\times 10^{6}\times n)/(∆t\times V)$$

*V* represents the volume of culture liquid in mL and *n* is the dilution factor.

### 4.4. Molecular Identification

Genomic DNA extraction was performed on the high-efficiency cellulose-degrading fungus using Gel DNA Extraction Ver. 4. 0 (TaKaRa, Shiga, Japan). PCR amplification was carried out using the GeneAmP 9700 system (Applied Biosystems, Foster City, CA, USA) with fungal primers ITS4 5′-TCCTCCGCTTATTGATATGC-3′ and ITS5 5′-GGAAGTAAAAGTCGTAACAAGG-3′ targeting the ITS (internal transcribe spacer) region [66]. Thermal cycle conditions were as follows: initial denaturation at 94 °C for 2 min, followed by 35 cycles of denaturation at 94 °C for 30 s, annealing at 55 °C for 30 s and extension at 72 °C for 60 s, and ultimately extension at 72 °C for 2 min. The amplified product was detected using a 0.8% agarose gel electrophoresis. The amplified product was then evaluated and deposited in the GenBank database (https://www.ncbi.nlm.nih.gov/datasets/genome/GCA_039906145.1/ (accessed on 19 January 2025)) under accession number MW88153 for further comparison and phylogenetic tree construction using the neighbor-joining method in the MEGA 11 software. The strains used to construct the ITS phylogenetic analysis are listed in Table 5. The phylogenetic relationship was inferred using this method, with bootstrap testing (1000 replicates) showing the percentage of replicate trees in which associated taxa clustered together above the branches. Genetic distances were computed using the Kimura 2-parameter method, and all positions containing gaps and missing data were eliminated [67].

The identified *Flavodon* sp. x-10 was deposited in the China Center for Type Culture Collection (CCTCC M2020961).

### 4.5. Genomic DNA Extraction and Sequencing

The high-quality genomic DNA was extracted and then subjected to an agarose gel electrophoresis to visualize genome integrity. Qubit was utilized for the accurate quantification of the genomic DNA concentration for further use. During library construction, the genomic DNA was fragmented using a 26GNeedle (SAI Infusion Technologies, San Diego, CA, USA) and fragments larger than 20 kb were selected with BluePippin (Sage Science, Beverly, MA, USA). Following end repair and A tailing, barcoded adaptors were attached to both ends of the fragments to create a DNA library. The resulting libraries were sequenced using the PacBio Sequel platform (MAGIGENE, Guangzhou, China), and concentration quantification and library quality inspection were carried out using Qubit (Thermo Fisher Scientific, Waltham, MA, USA).

### 4.6. Analysis of the Genomic Data

Genome size was estimated and evaluated using GCE (Genome Characteristics Estimation; version: gce-1.0.2) and k-mer frequency statistics. For fungal genome assembly, canu (v1.8) was utilized for three-generation assembly and error correction, pilon (v1.23) for two-generation error correction, and SSPACE (v1.1)-assisted blasr for sequence extension. Gene prediction was accomplished using the GeneMark (v1.14_1.25_lic) software, while tRNAscan-SE (v2.0.5), Barrnap, and tmRNA were employed for tRNA, rRNA, and Aragorn prediction in the genome, respectively. The LTR, LINE, SINE, as well as DNA transposons were analyzed using the RepeatMasker software (v4.1.2-p1), while tandem repeats were identified by the trf software (v4.10.0).

The predicted genes were functionally annotated in three databases, including GO, KEGG, and COG. The corresponding tool of CAZy database, hmmscan, was used to align non-redundant gene sets with CAZy (v2016.7.15), setting e-value ≤ 1 × 10^−5^ to obtain annotation information of the corresponding carbohydrate-active enzymes. TCDB is an auxiliary association database of biological transport systems, including sequence, function, category, structure, and evolutionary information. BLASTP against the genome with an e-value of 1 × 10^−20^ provided functional annotation information from TCDB database according to the relative abundance of the unigenes’ representative sequence.

## 5. Conclusions

In this study, the x-10 strain with strong cellulase activity was isolated from the litter of a poplar plantation and identified as *Flavodon* sp. through morphological and phylogenetic analyses. The genome sequencing and assembly results show that *Flavodon* sp. x-10 had a genome size of 37.10 Mb with 11,277 predicted genes, intermediate between the three cellulose-degrading fungi, *T. reesei*, *A. niger*, and *N. crassa*. The functional gene annotation of the whole genome of *Flavodon* sp. x-10 were performed, and 2080, 10,429, and 5039 unigenes were successfully annotated in the COG, GO, and KEGG databases, respectively. According to the further analysis of CAZy database, PCWDEs genes in *Flavodon* sp. x-10 were the most abundant (183), far more than those of the above three cellulose-degrading fungi. Compared to these fungi, the x-10 isolate also contains abundant AA coenzyme genes and has a more important degradation potential for lignocellulose, which is further demonstrated by its laccase activity assay.

## Figures and Tables

**Table 1:** Genome features of *Flavodon* sp. x-10.

Feature	Value
Raw bases	7,998,389,159
Raw reads	516,732
Assembled genome size (bp)	37,096,425
G + C content in assembled genome size (%)	49.48%
Protein-coding genes (CDS) (bp)	18,218,150
GC content in gene region (%)	52.28
Gene number	11,277
Average length of gene (bp)	1651
Gene/genome (%)	49.11
tRNA	157
rRNA	49
GO annotation	10,429
COG annotation	2080
KEGG annotation	5039
CAZy annotation	541
TCDB annotation	568

**Table 2:** Number of tRNA genes that transfer specific amino acids.

Amino Acids	Number of tRNA Genes	Amino Acids	Number of tRNA Genes
Ala	11	Arg	11
Cys	1	Gln	7
His	4	Ile	7
Met	5	Phe	4
Thr	8	Trp	2
Asn	3	Asp	7
Glu	12	Gly	13
Leu	10	Lys	8
Pro	8	Ser	13
Tyr	8	Val	11

**Table 3:** Repeat sequence count.

Repeat Sequence	Number of Elements	Length Occupied (bp)	Percentage of Sequence
SINEs	7	400	0.00%
LINEs	60	3739	0.01%
LTR elements	6	746	0.00%
DNA transposons	14	775	0.00%
Unclassified	1	42	0.00%
Small RNA	123	57,261	0.15%
Satellites	0	0	0.00%
Simple repeats	4067	179,908	0.48%
Low complexity	686	39,497	0.11%
Total		282,368	0.76%

**Table 4:** Number of annotated genes in each transporter family.

Transporter Family	Number
Translocating NADH Dehydrogenase (NDH) Family	19
The Cation Diffusion Facilitator (CDF)	7
Major Facilitator Superfamily (MFS)	103
Amino Acid–Polyamine–Organocation (APC) Family	23
Nuclear Pore Complex (NPC) Family	30
translocating F-type, V-type, and A-type ATPase (F-ATPase) Superfamily	24
Permease (ZIP) Family	10
The Retromer-dependent Vacuolar Protein Sorting (R-VPS) Family	12
Glycoside–Pentoside–Hexuronide (GPH): Cation Symporter Family	13
The Mitochondrial Carrier (MC) Family	17
The Membrane Attack Complex/Perforin (MACPF) Family	10
ATP-binding Cassette (ABC) Superfamily	38
The P-type ATPase (P-ATPase) Superfamily	19
The Endoplasmic Reticular Retrotranslocon (ER-RT) Family	11
The Drug/Metabolite Transporter (DMT) Superfamily	12
Fatty Acid Transporter (FAT) Family	24
The Autophagy-related Phagophore-formation Transporter (APT) Family	10
Others	186

**Table 5:** Strains used in the molecular phylogenetic analysis in this study.

Species	Voucher/Strain Number	Origin	GenBank Accession No.	Reference
			ITS	
x-10	——	China: Jiangsu	MW881531	In this study (CCTCC M2020961)
*Flavodon ambrosius*	BPI 893213	USA: Florida	NR_154000	[68]
*Efibula americana*	CFMR FP-102165	USA: Kentucky	NR_158394	[69]
*Efibula clarkii*	CFMR FD-228	USA: Massachusetts	NR_158395	[69]
*Ceriporia sordescens*	Miettinen 15492.2	USA	NR_148119	[70]
*Ceriporia humilis*	H V. Spirin 4706	Russia	NR_148120	[70]
*Ceriporia pierii*	‘pierii’ voucher Bernard Rivoire 1161	France	KX752604	[70]
*Ceriporia torpida*	H Murdoch 90	Finland	NR_158331	NA
*Ceriporia sericea*	H V. Spirin 4944	Russia	NR_148121	[70]
*Ceriporia albomellea*	BJFC Dai 15205	China	NR_158332	[71]
*Ceriporia variegata*	BJFC Li 1780	Unknown	NR_166794	[72]
*Trametopsis aborigena*	voucher Robledo 1236	Argentina	KY655336	[73]
*Flavodon subulatus*	voucher FLAS-F-63941	USA: Gainesville, Florida	MN997875	NA
*Flavodon* sp.	BAB-3601	India	KM066573	NA
*Flavodon* sp.	BAB-3680	India	KJ588863	NA
*Flavodon* sp.	BAB-3694	India	KJ588812	NA
*Flavodon* sp.	BAB-3697	India	KJ588815	NA
*Flavodon* sp.	BAB-3631	India	KJ670235	NA
*Flavodon* sp.	BAB-3977	India	KJ612016	NA
*Flavodon* sp.	BAB-3978	India	KJ612017	NA
*Flavodon* sp.	BAB-3997	India	KJ612048	NA
*Flavodon* sp.	BAB-4076	India	KJ670295	NA
*Flavodon* sp.	BAB-4774	India	KP686451	NA
*Flavodon* sp.	BAB-4912	India	KR155009	NA
*Flavodon* sp.	BAB-4961	India	KR155051	NA
*Flavodon* sp.	BAB-5032	India	KR155092	NA
*Flavodon* sp.	GNBP1	India	KT714246	NA
*Flavodon* sp.	Kasson 593A	USA	KU695251	[74]
*Flavodon* sp.	Kasson 593G	USA	KU695252	[74]
*Flavodon* sp.	M82	Mexico	KP096363	NA
*Flavodon* sp.	UFMGCB 1811	Mariana L.A. Vieira	HQ377275	[75]
*Cerrena aurantiopora*	NIBR FG0000102423 ITS	South Korea	NR_158290	NA

## Data Availability

The data presented in the study were deposited in DDBJ/ENA/GenBank under the accession JAGTUN000000000.

## Supplementary Materials

The following supporting information can be downloaded at: https://www.mdpi.com/article/10.3390/ijms26030866/s1.

## Abbreviations

PCWDE	Plant Cell Wall-Degrading Enzyme
LME	Lignin-Modifying Enzyme
MNP	Manganese-Dependent Peroxidase
LIP	Lignin Peroxidase
NCBI	National Center for Biotechnology Information
EG	Endoglucanase
CBH	CelloBioHydrolase
BG	*β*-Glucosidase
AA	Auxiliary Activity
TCDB	Transporter Classification Database
MFS	Major Facilitator Superfamily
KEGG	Kyoto Encyclopedia of Genes and Genomes
STs	Sugar Transporters
